# Canine leishmaniosis in South America

**DOI:** 10.1186/1756-3305-2-S1-S1

**Published:** 2009-03-26

**Authors:** Filipe Dantas-Torres

**Affiliations:** 1 Department of Veterinary Public Health, Faculty of Veterinary Medicine, University of Bari, 70010 Valenzano, Bari, Italy

## Abstract

Canine leishmaniosis is widespread in South America, where a number of *Leishmania *species have been isolated or molecularly characterised from dogs. Most cases of canine leishmaniosis are caused by *Leishmania infantum *(syn. *Leishmania chagasi*) and *Leishmania braziliensis*. The only well-established vector of *Leishmania *parasites to dogs in South America is *Lutzomyia longipalpis*, the main vector of *L. infantum*, but many other phlebotomine sandfly species might be involved. For quite some time, canine leishmaniosis has been regarded as a rural disease, but nowadays it is well-established in large urbanised areas. Serological investigations reveal that the prevalence of anti-*Leishmania *antibodies in dogs might reach more than 50%, being as high as 75% in highly endemic foci. Many aspects related to the epidemiology of canine leishmaniosis (e.g., factors increasing the risk disease development) in some South American countries other than Brazil are poorly understood and should be further studied. A better understanding of the epidemiology of canine leishmaniosis in South America would be helpful to design sustainable control and prevention strategies against *Leishmania *infection in both dogs and humans.

## Background

South America is a large continent located in the western hemisphere. Most of its landmass is situated within the tropical zone (which extends from the equator to the north and south parallels of 23°30'), which provides a very suitable environment for many kinds of arthropods (e.g., ticks, mosquitoes and phlebotomine sandflies) that can act as vectors of a number of pathogens. As a corollary, people living in South America are exposed to a number of arthropod-borne diseases, including malaria, leishmaniosis and dengue fever. Similarly, dogs are also affected by many arthropod-borne diseases, including ehrlichiosis, babesiosis, dirofilariosis and leishmaniosis [1,2].

Canine leishmaniosis is widespread in South America and it is among the most important canine vector-borne diseases occurring in this region, mainly because of its major zoonotic relevance [1-4]. The present article provides an overview on key aspects related to canine leishmaniosis in South America, emphasising future research needs.

## *Leishmania *species infecting dogs in South America

A number of *Leishmania *species have been isolated or molecularly characterised from dogs in South America (Table 1). They include *Leishmania amazonensis*, *Leishmania braziliensis*, *Leishmania colombiensis*, *Leishmania infantum *(syn. *Leishmania chagasi*), *Leishmania mexicana*, *Leishmania panamensis*, *Leishmania peruviana*, and *Leishmania pifanoi *[5-12]. With the exception of *L. amazonensis*, which has not been isolated from dogs so far, the other species have been isolated and characterised by traditional methods (e.g., isoenzyme electrophoresis) [5-11]. In the cases of canine leishmaniosis by *L. amazonensis *reported in São Paulo, south-eastern Brazil, the species identification was performed by using a *Leishmania*-specific rDNA-based PCR assay on lymph node samples, followed by hybridisation with a *L. amazonensis*-specific probe [12].

**Table 1:** *Leishmania *species infecting dogs in South America.

Species	Disease form	Suspected/proven vectors^a^	Geographical distribution
*L. amazonensis*	Visceral	Unknown	Brazil
*L. braziliensis*	Cutaneous	*Lu. whitmani*, among others	Argentina, Bolivia, Brazil, Colombia, Peru, Venezuela
*L. colombiensis*	Visceral	Unknown	Venezuela
*L. infantum*	Visceral	*Lu. longipalpis*, *Lu. evansi*, *Lu. youngi*, among others	Argentina, Bolivia, Brazil, Colombia, French Guiana^b^, Venezuela
*L. mexicana*	Cutaneous	*Lu. ayachuchensis*	Ecuador
*L. panamensis*	Cutaneous	*Lu. trapidoi*	Colombia, Ecuador
*L. peruviana*	Cutaneous	*Lu. peruensis*, *Lu. verrucarum*	Peru
*L. pifanoi*	Cutaneous	Unknown	Ecuador

^a ^*Lutzomyia *spp. that have been suspected to be involved in the transmission of *Leishmania *spp. to dogs in South America. Further information on the phlebotomine sand flies have been implicated as vectors of *Leishmania *spp. in this region can be found elsewhere [15,23].

^b ^Autochthonous transmission in French Guiana is uncertain (see text for details).

**Figure 1:**
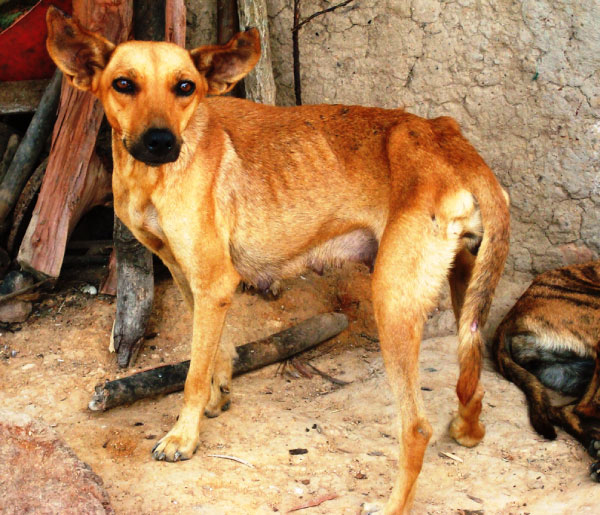
**Canine visceral leishmaniosis**. A *Leishmania*-seropositive dog showing facial muscular atrophy, skin lesions, loss of weight, and onychogryphosis.

*Leishmania infantum *is the most important causative agent of canine visceral leishmaniosis in South America. Dogs have been regarded as the main reservoir hosts of *L. infantum*, which is a parasite of major zoonotic concern, particularly in Brazil where ~3500 cases of human visceral leishmaniosis are reported annually; about 10% of the cases have resulted in a fatal outcome [13]. Dogs infected by *L. infantum *can develop a life-threatening disease characterised by lymphadenomegaly, muscular atrophy, skin ulceration, weight loss and onychogryphosis (Figure 1). It is a common concept that all dogs with visceral leishmaniosis in South America are infected by *L. infantum*. However, in Venezuela, a strain characterised by isoenzyme analysis as *L. colombiensis *was isolated from a dog presenting visceral leishmaniosis [7]. In Brazil, two dogs diagnosed as having visceral leishmaniosis were actually infected by *L. amazonensis *[12]. These reports highlight the importance of using proper diagnostic tools to identify the species of *Leishmania *involved in each case of canine leishmaniosis irrespective of the clinical form.

**Figure 2:**
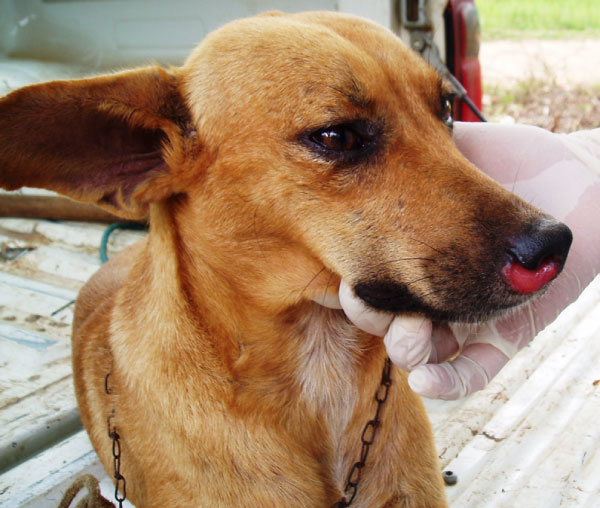
**Canine cutaneous leishmaniosis**. A *Leishmania*-seropositive dog presenting a mucocutaneous lesion on nose.

*Leishmania braziliensis *is the main causative agent of cutaneous leishmaniosis in dogs in South America [10]. Most of the dogs infected by *L. braziliensis *live in rural areas and they may present single cutaneous or mucosal lesions (Figure 2) [14]. Dogs have been suspected to play a role in the domestic transmission cycle of *L. braziliensis *and *L. peruviana *in some areas of South America, but there is only circumstantial evidence supporting this hypothesis [10]. In fact, the role of dogs in the maintenance of these parasites is probably minor [3].

Hybrid *Leishmania *strains have also been isolated from dogs in South America. For example, *L. braziliensis*/*L. peruviana *and *L. braziliensis*/*L. guyanensis *hybrid strains have been isolated from dogs in Peru and Venezuela, respectively [10]. The hybrid strains have phenotypic and genotypic features of two *Leishmania *species, and it has been suggested that these hybrids might represent strains that originated directly from a common ancestor or that they might be the result of genetic exchange [15].

In certain areas, the enzootic transmission cycles of different *Leishmania *parasites might overlap and dogs might become co-infected. For instance, cases of co-infection by *L. infantum *and *L. braziliensis *in dogs have been reported in south-eastern Brazil [16,17]. Co-infection by *L. infantum *and other trypanosomatids (e.g., *Trypanosoma evansi*) in dogs have also been reported [18]. For instance, a new species of *Trypanosoma *(namely *Trypanosoma caninum*) has recently been isolated from a dog co-infected with *L. braziliensis *in south-eastern Brazil [19]. Co-infections might be relevant in terms of diagnosis because of the possibility of serological cross-reactions among different *Leishmania *species [20] and with other related trypanosomatids [19,21].

## Transmission of *Leishmania *parasites to dogs

**Figure 3:**
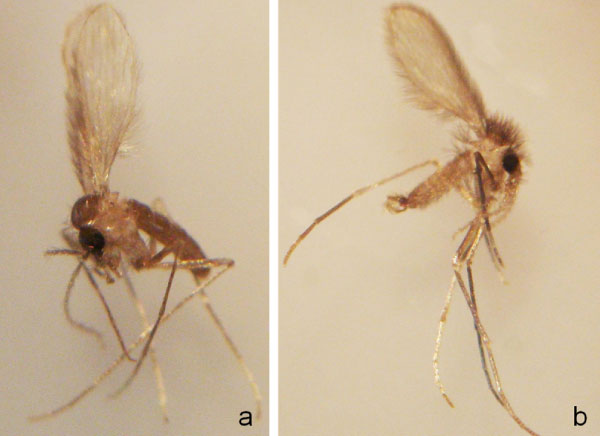
***Lutzomyia *sand flies**. A female (a) and a male (b) of *Lutzomyia migonei*, a putative vector of *L. infantum *in some regions of Brazil [24,32].

The primary mode of transmission of *Leishmania *parasites from dog to dog is through the bite of an infected phlebotomine sandfly. In South America, the vectors of *Leishmania *parasites belong to the genus *Lutzomyia *(Figure 3). Over 70 species of *Lutzomyia *have been suspected to be involved in the transmission of the eight *Leishmania *species known to infect dogs in South America [15,22-29]. However, the only well-established vector of *Leishmania *parasites to dogs in South America is *Lutzomyia longipalpis *[24]. This phlebotomine sandfly is a proven vector of *L. infantum*, widespread in South America [23], and has an opportunist feeding behaviour. However, it is interesting to note that in some areas dogs are not the preferred source of blood of *Lu. longipalpis*. For instance, in a recent study carried out in central-western Brazil, it was found that *Lu. longipalpis *fed preferentially on birds, rodents, humans, opossums, oxen, horses and dogs, in decreasing order of importance [30]. In these areas, the role of dogs as reservoir hosts of *L. infantum *could be of minor relevance.

Molecular biology techniques, in particular PCR-based tools, have impacted many fields of parasitology, including the study of a number of parasites and their respective arthropod vectors. The development of PCR-based tools for the detection of *Leishmania *DNA in phlebotomine sandflies has increased the number of putative vectors of *Leishmania *parasites in South America, see, for example, [25-29]. However, the detection of *Leishmania *DNA in a given *Lutzomyia *species, the mere detection *per se*, does not necessarily mean vector competence. In fact, experimental transmission studies are needed to prove the role of a given phlebotomine sandfly species as a vector of *Leishmania *parasites, even though these studies might be expensive, time-consuming and require considerable expertise on phlebotomine sandfly rearing.

The absence of *Lu. longipalpis *in some areas where cases of canine leishmaniosis have been reported [31,32] has suggested the participation of other phlebotomine sandfly species or the existence of secondary modes of transmission. Secondary modes of transmission that have been suggested in the literature include transplacental transmission [33], via blood transfusion [34], and venereal transmission [31,35]. However, the relevance of alternative ways of transmission is unknown. In a similar way, fleas and ticks have long been regarded as putative vectors of *L. infantum *in Brazil [36-39], but an overwhelming proof that they are competent vectors of *Leishmania *parasites has never been provided.

## Geographical distribution

**Figure 4:**
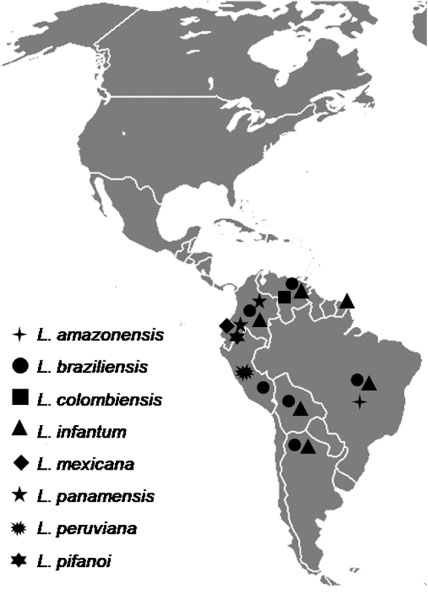
**Distribution of *Leishmania *spp. infecting dogs in South America**. The distribution of some species is probably wider than currently known. For instance, *L. braziliensis *and *L. infantum *are endemic in Paraguay [15], but there are no published reports of infection in dogs.

Canine leishmaniosis is a widespread disease in South America (Figure 4). Infection by *Leishmania *parasites in dogs have been reported in all countries except Chile, Uruguay, Suriname, and Guyana (see Table 1). Cases of canine visceral leishmaniosis by *L. infantum *(zymodeme MON-1) have been diagnosed in French Guiana, although autochthonous transmission has not yet been demonstrated [31].

*Leishmania braziliensis *and *L. infantum *are the most widespread species infecting dogs in South America and their distribution is probably wider than it is actually conceived. In recent years, there has been a southward spreading of canine leishmaniosis caused by *L. infantum *in South America. For example, the disease has recently been diagnosed in previously free areas of southern Brazil [40] and northern Argentina [41].

For a long time, canine leishmaniosis was considered to be a disease confined to rural areas. Nowadays, the disease is well-established in large urbanised areas such as the metropolitan region of Belo Horizonte, south-eastern Brazil [42]. Many factors could favour the spreading of canine leishmaniosis in South America, including the movement of dogs between endemic and non-endemic areas [31] and changes in vector ecology. *Lutzomyia longipalpis *is widespread in South America [23] and it is adapted to colonise environments modified by man [24]. In the State of Pernambuco, north-eastern Brazil, sparse spots of modified Atlantic rainforest can be found in highly urbanised areas. These remnants of Atlantic rainforest are potentially inhabited by phlebotomine sandflies of many species [43], including *Lu. longipalpis *[44]. It means that the introduction of a *Leishmania*-infected dog into a non-endemic area where the potential vectors are present could result in the establishment of a new focus of disease. In fact, if the current tendency continues [40,41], new foci of the disease should be expected to be detected in the future.

## Prevalence of infection

Most information on the prevalence of infection among dogs came from serological surveys conducted in Brazil, see, for example [45-48], and at a much lesser extent in other countries such as Argentina [49], Colombia [50] and Venezuela [51]. Although the prevalence of *Leishmania *spp. infection in dogs in South America can vary widely from region to region, and according to the diagnostic method used, it is usually over 25% [21,47,49,51,52] and might be as high as 75% in highly endemic foci [53]. However, it is difficult to estimate the overall prevalence of *Leishmania *infection in dogs in South America because of the limited amount of published data from some countries (e.g., Paraguay), the existence of methodological differences among studies (e.g., sample size and criteria of positivity) and the inherent limitations of serology (e.g., possibility of cross reactions).

An important epidemiological feature that has been observed in South America (and also in the Mediterranean basin) [54] is that the majority of the dogs infected by *L. infantum *are apparently healthy, exhibiting no visible clinical signs of visceral leishmaniosis. In some foci in Brazil, over 80% of the seropositive dogs might be clinically healthy [47,55]. This information might be relevant because seropositive but apparently healthy dogs can also serve as a source of infection to phlebotomine sandflies [56,57].

## Risk factors associated with infection and disease

**Figure 5:**
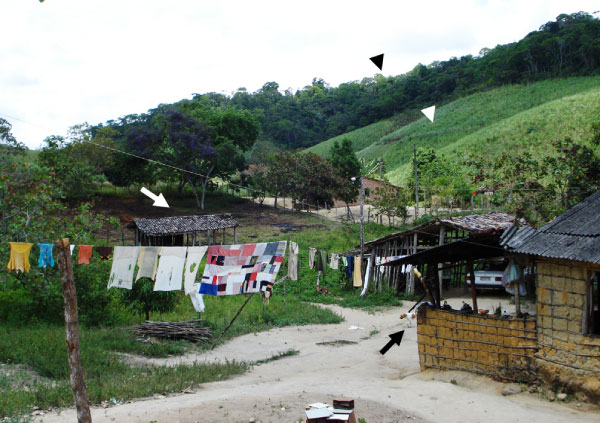
**Dog's lifestyle can increase the risk of *Leishmania *infection**. This picture shows a free-roaming dog (black arrow), an animal shelter (white arrow), a spot of modified Atlantic rainforest (black arrowhead), and an area of deforestation (white arrowhead) for agriculture. In this rural area, dogs are highly exposed to sand flies, which can be found inside houses, in animal shelters and forested areas.

**Figure 6:**
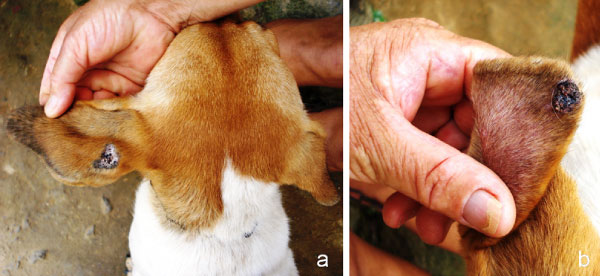
**Skin lesions in a *Leishmania*-seropositive dog**. Skin lesions on the ears of a dog, the same indicated in Figure 5, which was positive for anti-*Leishmania *antibodies. These lesions were possibly caused by *L. braziliensis*, which is highly prevalent in this area (municipality of Amaraji, Pernambuco, north-eastern Brazil) [59].

Studies attempting to assess the risk factors associated to infection in dogs in South America have been conducted mainly in Brazil. In some areas, there is a higher prevalence of anti-*Leishmania *antibodies among males when compared with females [47], but in others there has been no association between gender and seropositivity [48]. In some areas, there is a higher seroprevalence in young dogs (< 1 year) [47], whereas in other areas older dogs (1–6 years) are at a higher risk of infection [48]. This apparent disagreement among studies might reflect the local nature of canine leishmaniosis. The epidemiology of the disease varies widely among different regions and risk factors associated to infection in different disease foci might be difficult to predict. One important risk factor is the dog's lifestyle. For instance, guard dogs that are kept outside houses during the whole night are more exposed to sandfly bites and therefore are at a higher risk of infection as compared with companion dogs that are kept inside houses [48,58]. In some rural areas, dogs (Figures 5 and 6) are highly exposed to phlebotomine sandflies, which can be found inside houses, in animal shelters and forested areas [59].

The risk factors underlying the outcome of infection by *Leishmania *parasites in dogs in South America are poorly understood. The majority of the dogs living in rural and suburban areas are mongrel dogs [47] and the susceptibility to *L. infantum *infection in these dogs has been shown to be associated with MHC class II polymorphism [60]. The relationship between nutritional status (which is a known risk factor for human visceral leishmaniosis) and the susceptibility to *L. infantum *infection in dogs should be further investigated.

## Control of canine leishmaniosis in South America

### Vector control

Vector control is probably the most effective way to prevent *Leishmania *infection. For instance, a study conducted in Brazil has shown that deltamethrin-impregnated collars have potent anti-feeding and insecticidal effects on *Lu. longipalpis *and *Lutzomyia migonei *[61] and could reduce the risk of infection in dogs. The impact of this strategy within a community is dependent on collar coverage (*i.e*., number of dogs using the collar within a community) and loss rate [62]. In reality, the use of deltamethrin-impregnated collars is not very popular among dog owners living in rural and suburban areas, probably because of their costs. Usually, the poor social and economic conditions of many dog owners living in rural and suburban areas in South America do not allow them to afford even basic needs of life. Perhaps, a systematic control of phlebotomine sandflies in these areas, by using deltamethrin-impregnated collars or other strategies (e.g., spot-on combination of permethrin and imidacloprid) [63] could be possible, if supported by local public health authorities.

### Culling of seropositive dogs

While not universally accepted, the culling of seropositive dogs has long been recommended in Brazil [64]. However, in addition to be ethically arguable, the culling of seropositive dogs has had limited impact on the incidence of human visceral leishmaniosis. From 1990 to 1994, more than 80,000 dogs were culled in Brazil and during the same period there was an increase of almost 100% in the incidence of human visceral leishmaniosis [4]. The possible reasons (e.g., replacement of the culled dogs for susceptive puppies, low sensitivity and specificity of serological tests used to screen dogs to be culled, owners' unwillingness to cull their seropositive dogs) for the failure of this strategy have been extensively discussed in recent years [64-66]. One important feature that counts against this strategy is the fact that many culled dogs are not actually infected by *L. infantum*. In Rio de Janeiro (south-eastern Brazil), for example, a parasitological study of 66 dogs positive for anti-*Leishmania *antibodies revealed that 12 dogs were infected only by *L. braziliensis *[17]. In areas where both *L. infantum *and *L. braziliensis *are endemic, the use of contemporary techniques to identify the species involved in each case is imperative to avoid the culling of seropositive dogs that are actually infected by *L. braziliensis*.

### Vaccination

Until recently, there were no commercially available vaccines against canine leishmaniosis. Two vaccines have been licensed for use in Brazil. The first vaccine (Leishmune^®^, Fort Dodge Animal Health) consists of a *Leishmania donovani *glycoprotein fraction and presents 76–80% of efficacy [67]. The second vaccine (Leish-Tec^®^, Hertape Calier Saúde Animal) [68] consists of adenovirus expressing a *L. donovani *A2 antigen, but the results from phase-III trials have not been published yet. These vaccines are expected to become more and more popular among veterinarians and dog owners. Perhaps, the vaccination of dogs in association with a systematic vector control could replace the indiscriminate culling of seropositive dogs in endemic areas.

## Final considerations and research needs

Canine leishmaniosis is widespread in rural and urban areas in South America, although the factors associated with risk to *Leishmania *infection in dogs from this region are still poorly understood. Dogs are exposed to infection by a number of *Leishmania *species, which are potentially transmitted by different *Lutzomyia *species. Moreover, secondary modes of transmission might be involved and could be relevant for the establishment of new foci of canine leishmaniosis in non-endemic areas. Overall, this illustrates how complex is the epidemiology of canine leishmaniosis in South America and highlights the future research needs.

Little is known about the genetic relationship among the *Leishmania *parasites isolated from dogs, *Lutzomyia *sandflies and humans in many areas where canine leishmaniosis is endemic in South America. In this context, new attempts to isolate and characterise the species of *Leishmania *parasites circulating among dogs from urban and rural areas in different South American countries should be encouraged.

Despite of the long list of putative vectors, the only well-established vector of *Leishmania *parasites to dogs in South America is *Lu. longipalpis*. Indeed, dogs can serve as a source of *Leishmania *infection to different *Lutzomyia *species (e.g., *Lutzomyia whitmani*, *Lutzomyia evansi *and *Lutzomyia youngi*) [69-71]. However, it has yet to be proved that these *Lutzomyia *species are able to transmit the infection to a susceptible dog during a subsequent blood feeding.

In the same way, it is important to investigate the factors associated with risk to *Leishmania *infection in dogs, keeping in mind that these concepts cannot be generally extrapolated because canine leishmaniosis is a focal disease, whose epidemiology may vary widely from region to region. Some aspects (e.g., poor nutrition) might increase the risk of disease development, but so far this relationship has not been fully addressed in South American dogs. The factors dictating which dog will become sick (and when it will do so) should be addressed in future studies.

For some time, researchers working in South America have focused most of their efforts on canine leishmaniosis by *L. infantum *and *L. braziliensis*. Despite the inarguable importance of these two parasites, the study of canine leishmaniosis caused by other *Leishmania *species (e.g., *L. amazonensis *and *L. colombiensis*) would deserve more attention in the future. This constitutes a neglected issue that could provide new insights into the knowledge of the natural history of *Leishmania *parasites and the diseases they cause.

## Competing interests

The author declares that they have no competing interests.
